# *OpenCHIRP*: A Low-Cost, Lightweight Sub-Bottom Profiler for Shallow Water Environments Suitable for Autonomous Vehicles

**DOI:** 10.3390/s25237184

**Published:** 2025-11-25

**Authors:** Giuseppe Stanghellini, Fabrizio Del Bianco, Francesco Suriano, Luca Gasperini

**Affiliations:** 1 Institute of Marine Sciences, Italian National Research Council (CNR), 40129 Bologna, Italy; giuseppe.stanghellini@cnr.it; 2 Proambiente S.C.r.l., Via Piero Gobetti 101, 40129 Bologna, Italy

**Keywords:** *OpenCHIRP*, sub-bottom profiler, CHIRP, seismic reflection, shallow-water environments, pseudo-3D and 4D (repeated) seafloor and subseafloor seismic surveys

## Abstract

**Highlights:**

**What are the main findings?**

- Development of an innovative sub-bottom profiler for acquiring seismic reflection data in shallow-water environments.
- Key characteristics: low cost, lightweight, and compatible with autonomous vehicles.

**What are the implication of the main findings?**

- Extensive use of open hardware and software, with code and assembly instructions provided in the article.
- Enables seafloor and subseafloor pseudo-3D and 4D (repeated) mapping.

**Abstract:**

This paper presents the development of *OpenCHIRP*, an innovative sub-bottom profiler (SBP) designed for high-resolution seismic reflection surveys in shallow-water marine and lacustrine environments. The instrument employs chirped (frequency-modulated) impulses to penetrate the first few meters of unconsolidated sediments below the seafloor. Key characteristics include low cost, light weight, and low energy consumption, making it particularly suitable for deployment onboard Autonomous Surface Vehicles (ASVs). We discuss design, functionality, and potential applications of this innovative instrument, as well as results of the preliminary tests.

## 1. Introduction

CHIRP sub-bottom profilers (SBP) have evolved into essential tools for a wide range of underwater studies, including marine geology, archeology, and engineering [1,2,3]. The stability characteristics of the waves generated by these systems make them effective for estimating seabed reflectivity, which could enable quantitative assessments for classification of the seafloor [4,5,6].

CHIRP sonars are widely used in many different fields, dealing with marine geology, stratigraphic reconstructions, and assessment of sea- and lake-floor physical conditions, either for scientific purposes or for construction and detection of offshore infrastructures, e.g., [7]. They are the first and probably most diffused instrumentations onboard oceanographic vessels. Three-dimensional versions of the CHIRP-sonar system have been developed and successfully tested during marine geophysical surveys [8,9,10].

A novel and promising application of CHIRP-sonars to geo-forensic searches in shallow-water environment is described in Ruffell et al. [11], who applied a technological solution introduced by Bandini et al. [12], which consists of attaching a commercially available fishing sonar, via a winch mechanism, to an aerial drone.

Finally, innovative techniques based on the extensive use of AI open new perspectives to the effectiveness of CHIRP-sonar imaging, either for data processing [13,14] or interpretation [15,16].

Unlike impulsive systems, CHIRP-sonars transmit mechanical energy from electroacoustic transducers into the medium using a frequency-modulated (FM) wavelet instead of impulsive or time-gated monotonic waves. This distinctive approach allows for greater penetration while maintaining high vertical resolution, which primarily depends on the frequency content of the source signal.

The transmitted and received signals are used to deconvolve, correlate, or apply a matched filter to the data, effectively reducing uncorrelated noise between the two signals in the received data [17]. The relatively long outgoing sweep is compressed, and the final signal is transformed into a conventional seismic signal with an improved signal-to-noise ratio [18,19,20].

Chirped SBP systems are effective in very shallow water due to the reduced sensitivity of the frequency-modulated signal to reverberations. However, in extremely shallow water conditions (<5 m), the typical configuration of a coincident source and receiver may need modification, such as employing receiving transducers separate from the source. Thanks to their high resolution and seismic signal repeatability, CHIRP systems are invaluable for stratigraphic correlations and reflectivity estimates [21], as demonstrated by field analyses which can be performed using readily available open-source software packages such as ChirCor [22] and SeisPrho [23].

Typically, sweep sources in CHIRP sonars operate within a frequency range from a few hundred Hz to tens of kHz. Gutowsky et al. [24] tested seven sweep types with various envelope functions and instantaneous frequency functions to identify the optimal wavelets. To obtain time-series data reflecting the velocity–density function of the substrate and generate acoustic imaging for inferring geological properties, it is essential to remove the sweep using algorithms like cross-correlation or by applying a matched filter based on the autocorrelation of the source wavelet (Klauder wavelet).

Following this process, the data is often presented as the envelope function of the seismic signal [17], forming a new complex trace consisting of the original data (real part) and the quadrature-phase data obtained through the Hilbert transform (imaginary part). This relatively intricate process of acquiring and processing seismic data, often performed in real-time by specialized and high-performance electronic devices, makes traditional SBP systems expensive, inflexible, and somewhat limited in user interaction.

We have designed an innovative CHIRP sonar system called *OpenCHIRP*, specifically tailored for seismic reflection surveys in shallow-water environments. The standout features of this system include its lightweight design, low power consumption, and extensive use of “open-architecture” electronic devices and “open-source” software routines. This would imply a low cost of reproduction relative to available commercial systems. Moreover, *OpenCHIRP* could be equipped with “normal” audio waterproof emitting transducers, characterized in general by light weight. We anticipate that these characteristics will simplify the accessibility and understanding of this crucial tool among a broader community of scientists and pave the way for its integration into unmanned vehicles. Prototypes of *OpenCHIRP* were successfully tested onboard *OpenSWAP* [25] during multiple test sessions across various case studies, some of which are presented in this work.

## 2. Materials and Methods

At its core, a sub-bottom profiler functions in a relatively simple way: it generates a signal with specific length and frequency characteristics, emits it through a set of transducers at regular intervals (typically 4 to 16 times per second), and uses a hydrophone to receive the reflected signal from the underwater environment. The transducers are strategically placed just below the water’s surface and arranged to ensure that the emitted signal travels perpendicular to the seabed.

When the signal reaches the bottom, it experiences partial reflection and partial transmission. The transmitted portion penetrates deeper into the seabed, undergoing similar processes of partial reflection and transmission at each layer of varying density it encounters. This process allows the sub-bottom profiler to gather valuable information about the underlying layers of the seabed, including the geometry and terminations of reflectors, which can be used to infer details about depositional processes and environmental changes.

### 2.1. Hardware

Several models of SBP systems are available today, each fulfilling specific technical requirements based on the intended application (e.g., stratigraphy, archeology, environmental studies). Each SBP system features a custom hardware design that includes the generation of an impulse or wavelet, which is sent to a power amplifier and transducer coupled with the water column.

While current technologies make the development of a sub-bottom profiler appear deceptively simple, there are real-world challenges to address:

Signal Quality and Power: The generated signal must be of high quality and have sufficient power to penetrate adequately beneath the seafloor.
Hydrophone Sensitivity: The hydrophone must be sensitive enough to accurately capture the weak reflected signal from the seafloor.
Noise Reduction: The hydrophone receives not only the reflected signal but also ambient acoustic noise; effective methods are needed to mitigate this interference.

Autonomous Operation: For use with Autonomous Vehicles, the instrument must be compact, low-power, and self-contained, capable of performing data acquisition without reliance on an external, powerful computer.

Addressing these challenges ensures the sub-bottom profiler’s effectiveness and reliability in real-world applications, particularly for Autonomous Underwater Vehicles (AUVs).

To meet these technical specifications, we developed an all-in-one system incorporating as many open-hardware and open-source components as possible. The final system, described below, comprises an emitting system and an acquisition system. The acquisition system includes a compact computer that collects and stores data from the profiler in a standard format, geo-referenced with GPS-provided positions.

The *OpenCHIRP* system is designed to be compatible with unmanned systems, such as Autonomous Surface Vehicles like the *OpenSWAP* vehicles [25], allowing for complete programmatic control of its functions. By utilizing open-hardware and open-software components, we ensure flexibility, transparency, and ease of customization. The emitting system integrates smoothly with the acquisition system to facilitate accurate and reliable data collection.

A compact computer within the acquisition system is essential for managing and organizing the collected information, ensuring accessibility. Precise geo-referencing, facilitated by GPS integration, is critical for applications requiring spatial accuracy. Moreover, *OpenCHIRP*’s compatibility with autonomous systems represents a significant opportunity, particularly in environments where manual control is impractical or impossible. The ability to programmatically control all aspects of the system adds a layer of automation that enhances efficiency and reduces the need for human intervention.

Overall, this approach not only fosters innovation through the use of open technologies but also provides a robust and versatile solution for a wide range of applications.

**Figure 1:**
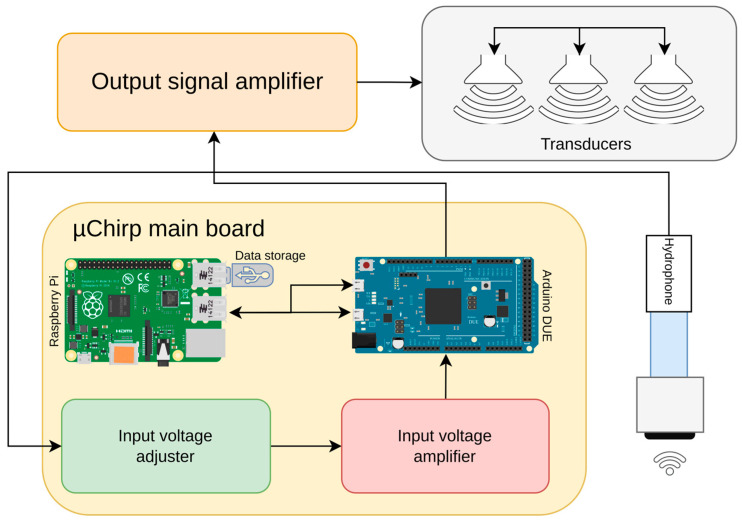
Hardware blocks of *OpenCHIRP* SBP system. The *Raspberry Pi* runs the data-logging program “**ciapciap**”, which records the measurements in **SEGY** format. An **Arduino Due** generates the chirped acoustic signal and captures the echoes reflected from the seabed and sub-seafloor layers. The acquired data is then sent to the Raspberry Pi for storage. The output signal amplifier, input voltage adjuster, and input voltage amplifier are all located on the electronic board shown in Figure 2, which also hosts both the Raspberry Pi and the Arduino Due.

The hardware blocks of the SBP system are displayed in Figure 1.

#### 2.1.1. Mainboard

By using open-hardware parts, maintenance is particularly straightforward in the case of problems or failures. Open-hardware components are typically well documented and widely supported by a community of developers and users. This accessibility ensures that replacements and repairs can be conducted efficiently and cost effectively, minimizing downtime and enabling interventions even during operations in remote locations.

Additionally, the modularity and standardization inherent in open-hardware designs facilitate easy upgrades and modifications. If a particular component becomes obsolete or a new, improved version is released, it can often be swapped out without requiring a complete overhaul of the system. This flexibility provides a significant advantage over proprietary systems, which may require proprietary parts and specialized knowledge for maintenance.

As shown in the block diagram of Figure 1, the system primarily consists of a Raspberry Pi connected to an Arduino DUE microcontroller. The Arduino DUE performs the following tasks:

- Receives, via its primary USB port, the start and end frequencies, the pulse length of the signal to emit, and the emission rate of the pulses.
- Builds the waveform to emit based on user parameters.
- Emits the waveform at the specified rate through one of its DAC ports.
- Simultaneously receives the signal from the hydrophone and sends it to its secondary USB port.

The Raspberry Pi performs the following tasks:

- Instructs the Arduino DUE on the signal to emit.
- Collects data sent by the Arduino DUE.
- Acquires real-time position data from a GPS device.
- Saves the geo-referenced data in a standard format on a USB disk.

To properly manage the emitted and received signals, the microcontroller requires signal conditioning electronics for input and output amplification.

**Figure 2:**
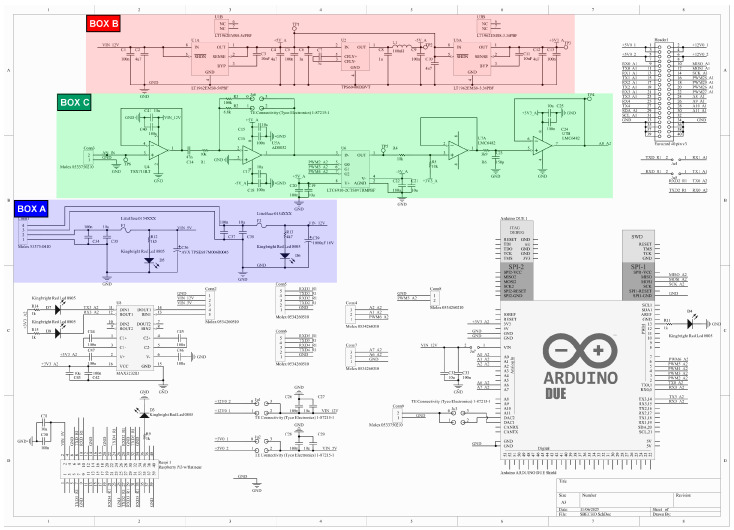
Schematic of the *OpenCHIRP* main board.

The Raspberry Pi, Arduino DUE, necessary voltage-processing electronics, and additional features are all arranged on the *OpenCHIRP* main board (Figure 2), designed using the free ECAD software *Altium CircuitMaker*.

The main features of this board are here briefly reported:

- Fused power supply equipped for external input of 5 and 12 V;
- Raspberry Pi and Arduino DUE slot for easy access and maintenance;
- Hydrophone signal processing: Custom voltage adjustment and gain amplifier programmable in real-time by the Arduino DUE;
- Expansion ports for additional integrations.

The *OpenCHIRP* main board schematic is reported in Figure 2, and includes three sections:

- A supply section (box A—Figure 2), designed to accept both 5 and 12 V on two separate rails and using a pre-crimped 4-pin connector. Decoupling and bypass capacitors are added, as well as interchangeable bottle fuse (default 3 A) and an SMD led for power-on tracking. No reverse polarity protection is provided in this board release.
- A specific low-noise regulated voltage section (box B—Figure 2), dedicated to the signal conditioning section (box C—Figure 2, described in iii.). This section, along with the connected signal conditioning part (iii.), ensures that interferences and noise affecting the hydrophone signal are kept as low as possible, thus enhancing the signal quality. The circuit design, along with the selection of components and integrated circuits (ICs) has been made to receive a signal from the hydrophone in the [6 ± 3] V range, and it can be easily reconfigured for different input signal ranges. Low-noise and low-dropout voltage regulators from LT technologies have been selected (for 5 V—IC U1A and 3.3 V—IC U3A) considering their market availability and their package (MSOP); the latter allows easy component swap and circuit reconfiguration. The IC U2 provides the −5 V voltage necessary for the proper signal conditioning (iii.). Passive components (C, R, L) are here used following manufacturers’ suggestions to make the regulation circuit work properly. Service test points are included on the PCB layout to check the regulated voltages.
- A conditioning section for the hydrophone signal (box C—Figure 2) that provides voltage adjustment and amplification of the input signal, labeled on the schematic as AN_IN, coming from a 2-pin pre-crimped connector. The hydrophone signal is first fed to IC U4, a single-channel operational amplifier (rail-to-rail and high voltage span, for potential system reconfiguration), used as a voltage buffer (for signal stabilization and impedance matching). It is connected to a first-order high-pass filter with a cutoff frequency of about 400 Hz. The filter and its capacitor block the DC offset, so the signal input to op-amp U5 is in the [0 ± 3] V range. U5, in inverting configuration, provides the first hardware signal amplification according to the JP6 jumper position. R2 and R3 resistors define the amplification factor, by default 0.68× (via R2) and 10× (via R3). Then, software amplification, controlled by the Arduino DUE board, is applied through IC U6: a programmable gain inverting amplifier (LTC6910, gain 0÷64, selected via 3 digital signals). The signal is then shifted around 1.65 V (op-amp U7A; U7 is a dual-channel, rail-to-rail, low-noise operational amplifier LTC6910) to ensure proper reading by the Arduino DUE Analog-to-Digital Converter. The shifted signal passes through a first-order low-pass filter (cutoff frequency about 52 kHz) and a voltage buffer (U7B) before being wired to the Arduino DUE at label A0_A2. Service test points are placed in section iii, as well as exposed ground pads to add a ground shield and reduce external interference. Bypass and decoupling capacitors are placed for all the ICs used.
- The Arduino DUE, Raspberry Pi, and additional connectors section (all parts not included in BOX A, B, and C—Figure 2) includes headers for easy maintenance and substitution of the Arduino and Raspberry Pi, as well as multiple connectors for customization and feature expansion. The available expansion ports are 1× RS232 port, 3× UART ports, 2× analog inputs (0–3.3 V), and 2 TTL digital I/O. Status LEDs are connected to the digital I/O of the Raspberry Pi and Arduino to provide information about the proper system operation. An additional header connector is also placed on the PCB to ensure compatibility with previous *OpenSWAP* hardware and for debugging purposes.

The components mounted primarily on the top of the board enable easy maintenance and part replacement, while the PCB form factor (Eurocard 10 × 16 cm with additional M3 mounting holes) provides numerous options for placing and securing the board.

**Figure 3:**
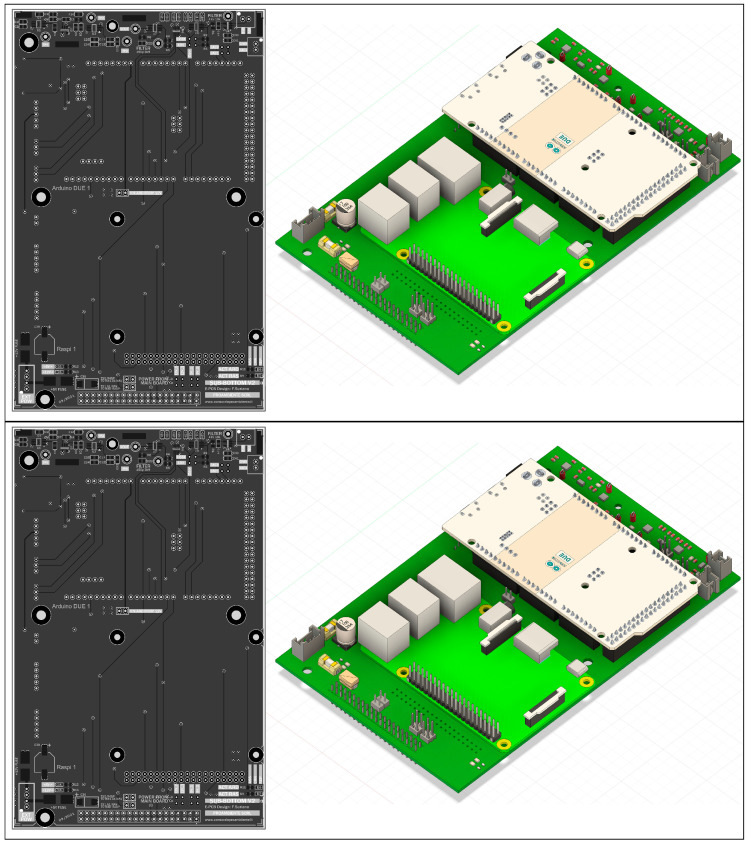
Main PCB of the OpenChirp SBP, which includes all components for the signal conditioning and I/O, as well as the connectors for Arduino and Raspberry boards.

This board will be released under an open-hardware license, and the routed PCB along with a 3D rendering of the board are shown in Figure 3.

#### 2.1.2. Seismic Reflection Signal Generation System

The system features a combined emitting and receiving electronic setup designed for generating and detecting seismic reflection signals from the sedimentary sequence. It operates using two different electronic configurations and types of transducers: one is a commercially available system, and the other is a custom-designed solution.

Commercial System: This configuration employs an off-the-shelf electronic driver paired with a standard underwater transducer. It is optimized for reliability and ease of integration, ensuring consistent signal output with minimal calibration needed.

Custom System: This second configuration consists of a specially designed electronic driver paired with a custom transducer. This system provides enhanced control over signal characteristics, such as frequency, pulse shape, and energy output, thereby optimizing performance for specific seismic exploration requirements.

Both systems are designed to produce controlled acoustic pulses that travel through the water and reflect off subsurface layers, allowing for high-resolution seismic imaging. The integration of commercial reliability with custom flexibility offers a versatile solution for underwater seismic studies.

All experiments utilized the same receiving unit, which consisted of a hydrophone and a preamplifier. However, two different emission units were employed, each comprising a transducer and an amplifier. The peripheral devices used in the tests are detailed in Table 1.

Table 1 displays the peripheral devices that were tested, along with the interconnection scheme for both testing configurations. It emphasizes the differences in the emission units while keeping the receiving setup consistent.

**Figure 4:**
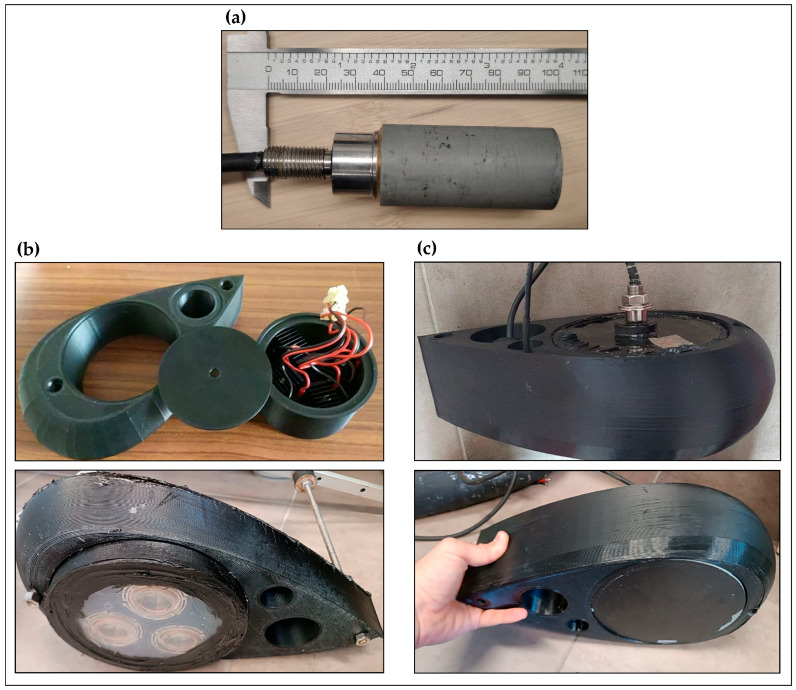
Prototypes of the transducer underwater case with the two transducers mounts. (**a**) The hydrophone used in both configurations. (**b**) The custom transducer. (**c**) The commercial transducer.

Both transmitter and receiver transducers were hosted in a hydrodynamic-shaped underwater case designed for the purpose of mounting the *OpenCHIRP* transducers onboard an Autonomous Surface Vehicle (Figure 4).

### 2.2. Software

The software system consists of two primary components: the Arduino DUE firmware, known as *OpenCHIRP*, and a Raspberry Pi program called *CiapCiap*. The firmware is developed in C++ and uploaded to the controller using the Arduino IDE, while *CiapCiap* is also written in C++ and operates on a Linux distribution, specifically Arch Linux, for the Raspberry Pi.

This paper does not provide a detailed analysis of the source code for these two programs but instead outlines their main features and usage. Readers can access the source code, as both programs are released under the GPL license.

Additionally, the software system includes the Arch Linux operating system running on the Raspberry Pi. It has been configured for unmanned operation, focusing on maximizing fault tolerance and enabling automatic shutdown and restart in the event of temporary malfunctions. This feature is especially important for deploying the instrument in Autonomous Vehicles.

#### 2.2.1. The Firmware

**Figure 5:**
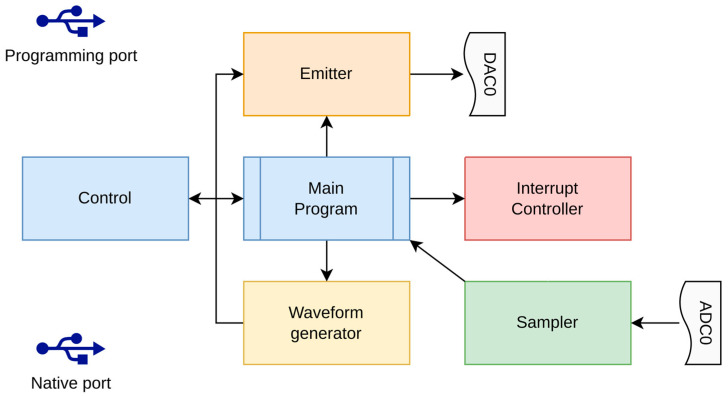
Block diagram of the OpenCHIRP firmware running on the Arduino Due. An interrupt-driven controller manages both the signal emission and the data acquisition, generating chirped waveforms while sampling the returning echoes. The transmitted signal is produced by the main firmware through a software-based waveform generator and output on **DAC0**, while the incoming echoes are captured via **ADC0**. Both **ADC0** and **DAC0** correspond to the respective ports of the **Arduino Due**. A dedicated control interface allows configuring the **Arduino Due**, specifying what signal to emit and when to emit it.

This software operates within an Arduino DUE microcontroller. The Arduino DUE features two USB ports: one designated as the “Native port” and the other as the “Programming port”. The “Native port” is significantly faster and is therefore utilized for receiving the acquired data back. Meanwhile, the other USB port is employed for transmitting commands and parameters to the Arduino to configure the low-level acquisition process. Figure 5 illustrates the block diagram of the program.

Two additional ports of the Arduino DUE are utilized:

- The DAC0 (Digital Analog Converter) port for emitting the waveform towards the amplified transducers.
- The ADC (Analog Digital Converter) A0 port for sampling the signal received by the hydrophone.

Upon startup, the program flow enters an infinite loop, awaiting commands. Although the command set is small, it enables control over all necessary aspects to program the emission and acquisition processes. The commands and parameters are listed in Table 2.

#### 2.2.2. The CiapCiap Program

**Figure 6:**
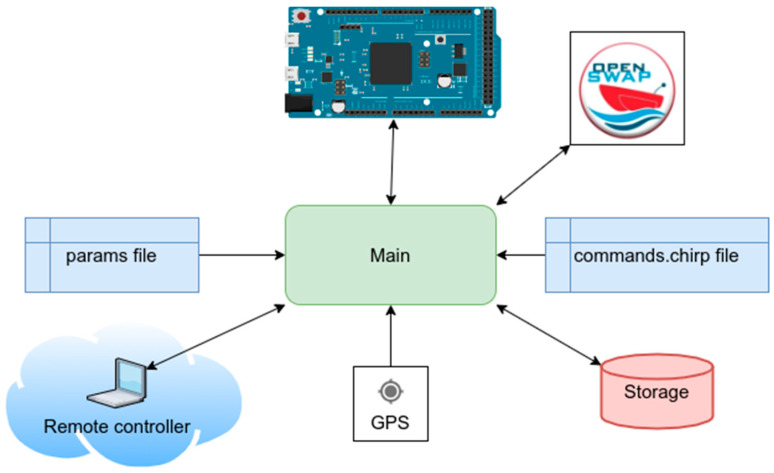
Block diagram of acquisition program *CiapCiap*. The software loads the param and commands.chirp configuration files, which define the input/output settings and the signal-generation parameters. Based on these settings, it instructs the Arduino Due on how to perform the emission sequence. The program receives data simultaneously from the GPS module and from the Arduino Due, then stores the georeferenced measurements in SEGY format. The entire acquisition and emission process can be controlled either by a remote PC or by an *OpenSWAP* autonomous platform.

It is the main software used for acquisition and control, providing extensive management of the emission and acquisition processes through two configuration files that contain commands and parameters for the Arduino DUE microcontroller. This program starts automatically during the Raspberry Pi’s boot sequence. It reads the two configuration files, “params” and “commands.chirp,” and then begins the acquisition process. The software’s block diagram is shown in Figure 6, while the block architecture is displayed in the Supplementary Materials.

The *CiapCiap* code is entirely configured through these two files, devoid of any graphical user interface. However, this absence of a GUI should not pose an issue, given that the program is intended for use in remote vehicles. Upon startup, the program searches for a file named “params” in the current directory, thereafter configuring all operations based on the contents of that file. Although the block diagram appears simple, the software itself is quite complex. It requires time to thoroughly explore the parameters in the configuration files and understand their functions. The full source code of the *CiapCiap* program is available at https://gitlab.com/giuseppe.stanghellini/ciapciap (accessed on 22 October 2025).

#### 2.2.3. The “Params” File

This file is structured in a position-dependent manner, where each individual line is designated to specify a distinct parameter. *CiapCiap* starts the reading process with the expectation that each line will consistently contain the correct parameter. Consequently, if there are any lines missing, *CiapCiap* will encounter problems, resulting in the file not being interpreted correctly. To elaborate, each line is composed of values that are intended to be assigned to the parameter corresponding to that specific line. These values are followed by one or more spaces and, optionally, a ‘#’ character. Should this character be present, it serves as an indicator that any subsequent characters extending to the end of the line are to be disregarded and treated as non-executable comments by *CiapCiap*. In Appendix A, a line-by-line description of the parameters file is reported.

#### 2.2.4. The Hosting Operating System

The Raspberry Pi running the acquisition jobs uses an Arch Linux distribution for ARM microprocessors. The installation is minimal, not requiring any desktop environments or graphical user interfaces. It is configured to auto-login as the root user and starts all services and acquisition processes. System images are freely available for download.

The system is configured entirely by configuration files inside the /boot/OPENSWAP folder. This folder is mounted read-only to preserve it in case of unexpected reboot/freeze of the system. All file systems, except /DATA, are mounted read-only to preserve their integrity. To modify files on those file systems, a five-step sequence is required:

(1) Login by SSH to OpenCHIRP.
(2) Execute RW.SH to make the folder writable.
(3) Edit the files for acquisition parameters.
(4) Kill ciapciap.
(5) Execute RO.SH to make the folder read-only again.

#### 2.2.5. The Boot Process

When powered on, the Raspberry Pi automatically logs in as the root user, causing the profile file inside the /root folder to be executed by the bash shell. This file executes the /boot/OPENSWAP/autostart.sh script, which spawns several processes and runs the following shell scripts:

- prologue.sh: Prepares the execution environment, sets global environment variables, and checks existing file systems for consistency.
- start_net.sh: Starts the network and configures the access point.
- start_acq.sh: Starts the acquisition process.

## 3. Results

**Figure 7:**
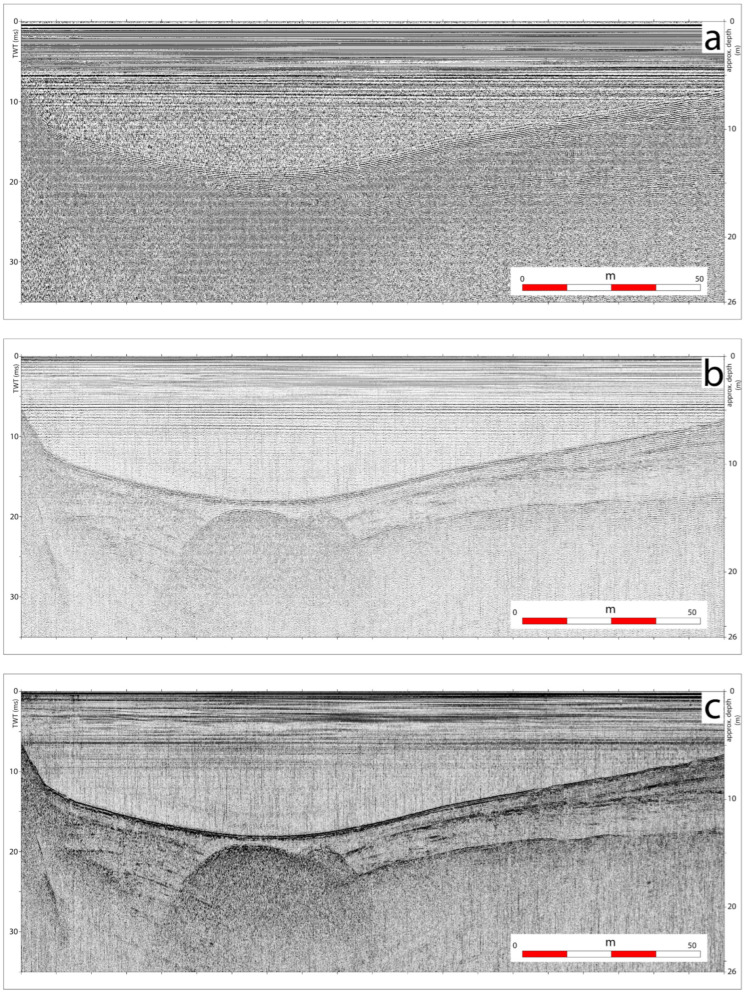
Example of high-resolution seismic reflection profile collected in an Alpine Lake (Lake of Cavazzo, NE Italy) using the *OpenCHIRP* system mounted onboard an *OpenSWAP* vehicle. (**a**) Raw data; (**b**) after de-chirping; (**c**) after application of an instantaneous amplitude correction.

In order to test the performance of *OpenCHIRP*, we installed it onboard an Autonomous Surface Vehicle of the *OpenSWAP* class and carried out several test sessions in a small, shallow-water alpine lake, the Lake of Cavazzo, during geo-environmental studies [26]. This enabled us to perform a fine-tuning of source and receiver configurations, and test the optimal parameters, such as geometry of the transducers, sweep length, and operating frequency range. Figure 7 displays an example of the seismic reflection profiles collected in about 20 m of water depth in the Lake of Cavazzo.

It shows a penetration of the signal reaching over 10 m below the lake’s floor, and includes three main steps of data processing, including the raw data plot (Figure 7a), which shows a low signal-to-noise ratio due to the presence of environmental noise and the absence of de-chirp processing applied to the data. Figure 7b shows the same section but after de-chirping and allows us to identify seismic reflection with centimetric resolution. Typical representation of the CHIRP sub-bottom profile is in the instantaneous amplitude of the signal, as shown in Figure 7c.

We then performed some tests using two different emitter/receiver configurations.

### 3.1. Setup-1

In the initial phase of testing, the emitting transducer consisted of three sound exciters (see Table 1) connected in series and driven by a 1500 W Class D amplifier. This configuration operated within a frequency range of 1 kHz to 10 kHz, adhering to the performance limits of the amplifier–transducer assembly. Under these conditions, a frequency-modulated “chirped” pulse ranged between 2 and 7 kHz.

### 3.2. Setup-2

The second phase of testing employed a commercially available piezoelectric transducer designed for underwater applications (see Table 1). This transducer was driven by dedicated control electronics, which functioned as both an impedance transformer and an amplification stage. In this configuration, the system operated at higher frequencies, ranging from 10 kHz to 25 kHz. Within this range, two different modulation settings were applied to the emitted pulse: one between 10 kHz and 15 kHz and the other between 20 kHz and 25 kHz.

**Figure 8:**
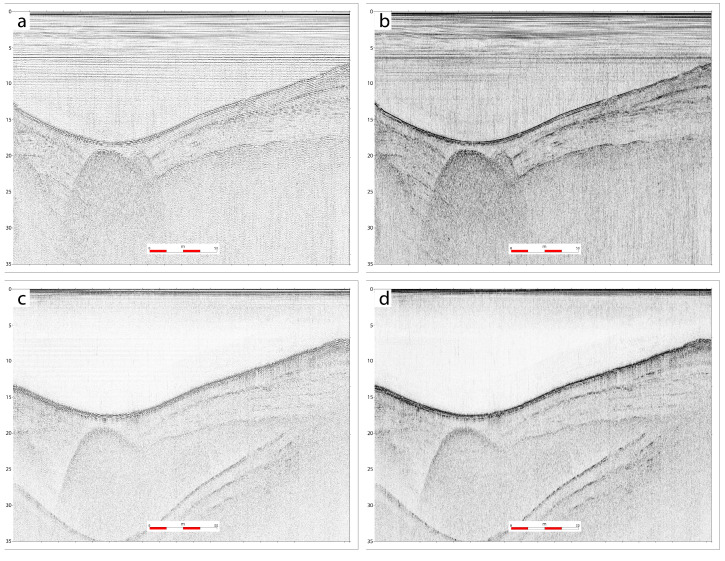
(**a**,**b**): Seismic reflection profile collected using Setup-1, before (**a**) and after (**b**) application of instantaneous amplitude correction; (**c**,**d**): same profile collected using Setup-2.

Figure 8 reports results obtained using the two configurations, Setup-1 and -2, which led to obtaining comparable results both in terms of vertical resolution and penetration of the seismic signal.

We note that the different setups allow for detecting similar stratigraphic information, although Setup-1 shows a deeper penetration of the signal. Conversely Setup-2 shows a higher resolution in the upper part of the section due to the higher frequency content.

**Figure 9:**
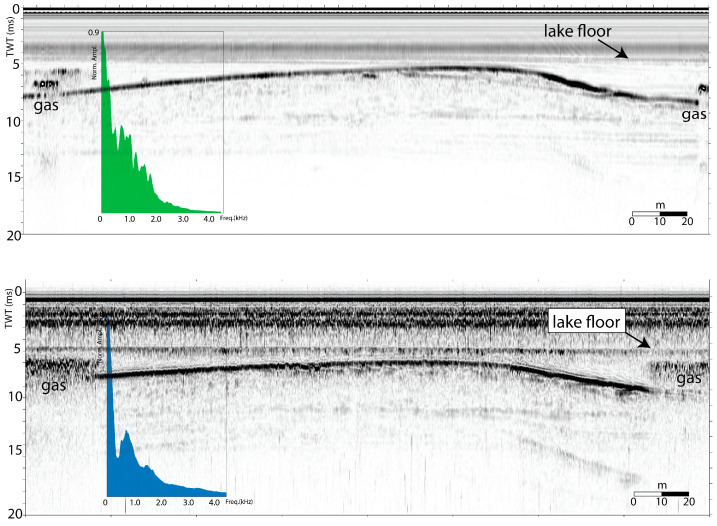
An example of seismic reflection profiles along the same navigation line collected with two different systems in Lake Trasimeno (Central Italy). (**Top**): CHIRP III Teledyne-Benthos, with 4 Massa transducers; (**bottom**): *OpenCHIRP* in configuration *Setup-1*. Unconsolidated sediments are penetrated down to 15–20 ms TWT below the lake floor, by both systems, with high vertical resolutions (tens of cm), enabling a detailed imaging of the sedimentary sequence. Quantitative comparison of the two systems could be performed through analysis of the frequency contents (insets) which appear similar, although *OpenCHIRP* shows the energy more focused on the higher part of the spectrum.

We also performed a comparison between a commercial system, i.e., the Teledyne Benthos CHIRP III sub-bottom profiler with our *OpenCHIRP*, in imaging the shallow subsurface in Lake Trasimeno (Central Italy) during a seismostratigraphic survey [27]. Figure 9 displays results of this comparison, which led to similar performances regarding penetration and resolution. But while the CHIRP III system was operated using a normal boat due to its dimensions and energy consumption, *OpenCHIRP* operated onboard an *OpenSWAP* vehicle.

The availability of CHIRP-sonar systems that could be employed onboard Autonomous Surface Vehicles opens the door to pseudo-3D and 4D (repeated) seismic reflection and bathymetric surveys of shallow-water environments, such as coastal lagoons, beaches, and lakes, and could contribute to quantitatively evaluate the effects of anthropic and natural pressures. Moreover, the low cost and the open architecture of *OpenCHIRP* might enlarge the potential community of users, including Universities, Oceanographic Institutes, and Environmental Agencies, which find in the relatively high costs of purchase and maintenance of such devices a great obstacle that can be rarely overcome.

## 4. Conclusions

This study presented a successful design, implementation, and testing of a novel sub-bottom profiler (SBP) optimized for high-resolution seismic reflection surveys in shallow-water environments. By employing CHIRP (frequency-modulated) impulses, the developed SBP achieves effective sediment penetration while maintaining a compact, lightweight, and energy-efficient design. These characteristics make it an ideal instrument to be integrated with Autonomous Surface Vehicles (ASVs), enhancing their capability to perform detailed subsurface investigations in both marine and lacustrine settings.

Results of the preliminary tests confirm the SBP’s efficacy in achieving high-resolution subsurface imaging, validating the core design principles and operational goals. Its low-cost and energy-efficient features ensure accessibility and broad applicability, paving the way for widespread adoption in geophysical research, environmental monitoring, and resource exploration.

Furthermore, the comprehensive details provided in this paper ensure that the SBP can be reproduced and adapted to suit diverse research needs. By sharing the design and operational insights, this work invites further innovation and optimization by the broader scientific community.

In summary, the development of this innovative SBP represents a significant step forward in seismic survey instrumentation. It addresses critical needs for cost effectiveness, portability, and compatibility with modern unmanned systems. Future work will focus on expanding its capabilities, such as enhancing sediment penetration depth and exploring additional functionalities, to further broaden its application potential. This advancement underscores the potential for innovative engineering solutions to transform the field of shallow-water seismic surveys.

## Figures and Tables

**Table 1:** Different emitter–receiver configurations tested during the development of *OpenCHIRP*.

Device	Brand	Operating Frequencies	Main Features
Hydrophone	Benthowave BII-7016	5 kHz–30 kHz	Program. Gain Preamp: 0, 20, 40, 60 dB. Omnidir. X 230°
Custom transducer	Dayton Audio sound exciter	100 Hz–10,000 Hz	40 W RMS-4 OHM
Custom driver	Sure-Electronics Audio Amplifier Board	20 Hz–20,000 Hz	1500 W @ 2 Ohm 84 V DC Class-D Amplifier
Commercial transducer	Benthowave BII-7506/22	Res. Freq. 23.4 kHz	Conical beam pattern 25° beam width @fs
Commercial driver	BenthowaveBII-5065 Amp.	135 Hz–60 kHz @ + 60 VDC	260 W @ + 60 VDC

**Table 2:** Commands and parameters controlling data acquisition.

Command
START = initial_frequency: the initial frequency of the generated FM waveform in Hz.
END = final_frequency: the final frequency of the chirped generated waveform in Hz.
DURATION = length: the duration in microseconds of the generated waveform.
FREQ = rate: the emission rates of the waveforms in Hz.
MKSIGNAL: generate the signal according to values above.
GO: starts the emission and acquisition process.
STOP: stops signal emission and acquisition process.

The source code of the *OpenCHIRP* firmware is available at https://gitlab.com/giuseppe.stanghellini/uChirp (accessed on 22 October 2025).

## Data Availability

The data presented in this study are openly available in the GitLab repository at uChirp, accessible at: https://gitlab.com/giuseppe.stanghellini/uChirp (accessed on 20 October 2025).

## Supplementary Materials

The following supporting information can be downloaded at https://www.mdpi.com/article/10.3390/s25237184/s1.

## Abbreviations

The following abbreviations are used in this manuscript:

ASV	Autonomous Surface Vehicle
SBP	Sub-bottom Profiler

## Appendix A

1. **NAME/TYPE STRING**: The name of the instrument, typically CHIRP.
2. **INPUT UDP PORT/TYPE NUMERIC**: The port to listen for incoming commands. An external program can send commands to CiapCiap to control the acquisition process.

- **UDP PORT OF MONITORING APP/TYPE NUMERIC**: The port to send received data for remote monitoring. When CiapCiap acquires new data, it stores it in SEGY format inside the internal storage device and then sends a subset of that data to a listening application over the Internet (or intranet).

3. **TCP/IP ADDRESS OF MONITORING APP/TYPE IP_ADDRESS**: The IP address of the host running the monitoring application.

- **TRACES DECIMATION/TYPE NUMERIC**: The traces decimation factor. This parameter specifies how many of the traces collected during the acquisition process must be sent to the listening application over the Internet, allowing to limit the outgoing traffic.
- **DATA DECIMATION/TYPE BOOLEAN**: The data decimation factor. This parameter allows for further reduction in the data sent over the Internet. If the value 1 is specified, the trace will be resampled to 512 bytes before sending it.

4. **DATA FOLDER/TYPE STRING**: The directory where SEGY files are stored.

- **FILENAME PREFIX/TYPE STRING**: The prefix of the filename of the stored data. The full filename includes a date stamp, time stamp, and the suffix “.SEG.”

5. **SPLIT EVERY BYTES/TYPE NUMERIC**: The maximum allowed size in bytes of a file.

- **μChirp COMMANDS FILE/TYPE STRING**: The filename containing commands to set the μChirp parameters.

6. **UNUSED**.
7. **UNUSED**.

- **DUNVAG SERIAL DEVICE/TYPE PATH**: The device to which the *OpenSWAP* vehicle is connected. Used when installed inside an *OpenSWAP* Autonomous Vehicle.
- **BAUD/TYPE NUMERIC**: The baud rate of the serial connection to Dunvag.

8. **DATA BITS/TYPE NUMERIC**: The data bits of the serial connection to Dunvag.
9. **PARITY/TYPE CHAR**: The parity of the serial connection to Dunvag.
10. **STOP BITS/TYPE NUMERIC**: The stop bits of the serial connection to Dunvag.
11. **GPS SERIAL DEVICE/TYPE PATH**: The device to which the GPS is connected.

- **BAUD/TYPE NUMERIC**: The baud rate of the serial connection to GPS.

12. **DATA BITS/TYPE NUMERIC**: The data bits of the serial connection to GPS.
13. **PARITY/TYPE CHAR**: The parity of the serial connection to GPS.
14. **STOP BITS/TYPE NUMERIC**: The stop bits of the serial connection to GPS.
15. **BOTTOM TRACKING START SEARCH/TYPE NUMERIC**: The starting point to search the bottom in ms. This parameter must be set to −1 for sub-bottom operation to disable bottom-tracking.
16. **STEP_FUNCTION_LENGTH/TYPE NUMERIC**: The step function length in the number of samples to use for bottom tracking. Unused for sub-bottom operation. 25–30. **Unused Parameters** for bottom tracking.
17. **GPS PORT/TYPE NUMERIC**: The port at which to start a GPS service.
