# Supplementary material for: OpenCHIRP: A Low-Cost, Lightweight Sub-Bottom Profiler for Shallow Water Environments Suitable for Autonomous Vehicles
Source: Sensors (Basel). 2025 Nov 25;25(23):7184. doi: 10.3390/s25237184 (PMC12694232; doi:10.3390/s25237184)
Supplement: Supplementary file 1 [file sensors-25-07184-s001.zip › sensors-3967660-supplementary.pdf]

Supplementary Material

Ciapciap Block Architecture Description

Acquisition, Merge, and Storage Block

- Initializing and configuring the acquisition hardware based on parameters received from the networking block.
- Continuously sampling sensor signals at the configured rate.
- Reading the latest available positioning data from the positioning block.
- Performing time alignment and merging of the sensor data with the position/georeferencing information.
- Storing the merged results into local files using the selected output format.
- This block suspends acquisition, reinitializes hardware, and resumes processing when parameters are updated.
- Shared positioning data is protected via synchronization to prevent inconsistent reads.

Positioning Block

- Continuously reading NMEA or binary positioning messages from the GPS receiver.
- Parsing and validating incoming data.
- Updating the shared positioning structure used by the acquisition block.
- Protecting updates with a mutex or equivalent synchronization primitive.
- Operates independently of acquisition timing.

Networking and Remote Control Block

- Receiving commands over TCP/UDP from a remote controller or mission-planning system.
- Processing configuration commands such as updating parameters, pausing/resuming acquisition, and clean shutdown.
- Sending status messages and acknowledgments to the remote controller.
- Propagating parameter updates and control flags to the acquisition block.
- Uses atomic updates, requiring no synchronization with positioning data.

Inter-Block Synchronization and Communication

- Positioning data structure shared between the positioning and acquisition blocks, protected by a mutex.
- Parameter set updated by networking, read by acquisition with an update flag.
- Control flags used for pause/resume, shutdown, and reinitialization requests.

Overall System Behavior

- The positioning block continuously refreshes the GPS data.
- The acquisition block collects sensor data, merges it with GPS data, and stores the results.
- The networking block receives remote commands and manages dynamic reconfiguration.
